# Comparison of Select Analytes in Exhaled Aerosol from E-Cigarettes with Exhaled Smoke from a Conventional Cigarette and Exhaled Breaths

**DOI:** 10.3390/ijerph111111177

**Published:** 2014-10-27

**Authors:** Gerald A. Long

**Affiliations:** Lorillard Tobacco Company, P.O. Box 21688, Greensboro, NC 27420, USA; E-Mail: glong@lortobco.com; Tel.: +1-336-335-6607; Fax: +1-336-335-6640

**Keywords:** smoking, vaping, electronic cigarette, e-cigarette, aerosol, carbonyl, phenolic, hydroxybenzene, combustion, nicotine, emission, passive vaping

## Abstract

Exhaled aerosols were collected following the use of two leading U.S. commercial electronic cigarettes (e-cigarettes) and a conventional cigarette by human subjects and analyzed for phenolics, carbonyls, water, glycerin and nicotine using a vacuum-assisted filter pad capture system. Exhaled breath blanks were determined for each subject prior to each product use and aerosol collection session. Distribution and mass balance of exhaled e-cigarette aerosol composition was greater than 99.9% water and glycerin, and a small amount (<0.06%) of nicotine. Total phenolic content in exhaled e-cigarette aerosol was not distinguishable from exhaled breath blanks, while total phenolics in exhaled cigarette smoke were significantly greater than in exhaled e-cigarette aerosol and exhaled breaths, averaging 66 µg/session (range 36 to 117 µg/session). The total carbonyls in exhaled e-cigarette aerosols were also not distinguishable from exhaled breaths or room air blanks. Total carbonyls in exhaled cigarette smoke was significantly greater than in exhaled e-cigarette aerosols, exhaled breath and room air blanks, averaging 242 µg/session (range 136 to 352 µg/session). These results indicate that exhaled e-cigarette aerosol does not increase bystander exposure for phenolics and carbonyls above the levels observed in exhaled breaths of air.

## 1. Introduction

Electronic cigarettes (e-cigarettes) are products that became available to United States consumers in about 2007 [1]. Unlike conventional cigarettes that burn tobacco at high temperatures, e-cigarettes contain a liquid flavor solution (e-liquid) that is thermally vaporized by a battery powered heating element. The e-liquids typically contain a mixture of aerosol forming components such as glycerin and propylene glycol, various flavors and, optionally, nicotine. Recently published studies have reported on the constituents of e-liquids and e-cigarette aerosols [2,3,4,5,6,7,8]. Some of these constituents are among those listed as Harmful and Potentially Harmful Constituents (HPHC) for tobacco products by the United States Food and Drug Administration (FDA) [9]. Constituents that have been identified in machine-generated e-cigarette aerosols and emissions in enclosed spaces [3,4,6,10], include the carbonyl compounds acetaldehyde, acrolein and formaldehyde [3,6,11,12]. The reported levels of these carbonyl compounds were lower than those of conventional cigarettes smoked under comparable conditions by one to two orders of magnitude.

Riker, *et al.* have advanced the notion that exhaled e-cigarette aerosol may pose an exposure risk to bystanders similar to that of environmental tobacco smoke (ETS) from conventional cigarettes through “passive vaping” [13]. However, the majority (~85%) of ETS aerosol arises from side stream smoke generated during static cigarette smolder in between puffs [14], which is absent for e-cigarettes. Several investigators have reported machine generated e-cigarette aerosol contributions to particulates/droplets and chemical constituents in test chambers [13,15] and indoor environments [5]. All of these studies suggest that exposure to constituents in machine-generated mainstream e-cigarette aerosols would not exceed background, although such studies did not actually use exhaled e-cigarette aerosol from human subjects.

Recent investigations have reported emissions of constituents in closed air chambers or in rooms having minimal ventilation with human subjects using e-cigarettes [15,16,17,18]. A study by Romanga, *et al.* in an unventilated room using human subjects failed to detect a number of analytes including nicotine [16], consistent with the sampling and analytical challenges posed by the baseline levels of many of the constituents in e-cigarette aerosols.

A 2013 study by Schripp, *et al.* reported aerosol droplet counts and chemical constituents generated by e-cigarette users, under prescribed puffing parameters, in a room with air exchange [17]. Several compounds, including carbonyls, were detected. However, the authors attributed these levels to the test subjects’ normal metabolic processes and not to the exhaled e-cigarette aerosols.

A recent study with nine e-cigarette users puffing *ad libitum* in a room with air exchange found propylene glycol, glycerin and nicotine in the room air [18]. No increases above background were noted for formaldehyde, acetone or acrolein.

These studies have explored the potential for bystander exposure from e-cigarettes, but that have not adequately addressed the chemical composition of exhaled e-cigarette aerosol. A simple mass balance and distribution of known constituents such as water, glycerin and nicotine has not been reported for exhaled e-cigarette aerosol. The quantities of constituents such as phenolics and carbonyls in exhaled cigarette smoke relative to exhaled e-cigarette aerosol, and to a suitable blank of exhaled breaths of air is also lacking in the scientific literature. The present study addressed these gaps with direct analyses of the quantities of phenolic and carbonyl compounds in the exhaled aerosols from human subjects using cigarettes and e-cigarettes without any dilution effects due to room volume or air exchange and determined mass balance and distribution of water, glycerin and nicotine in exhaled e-cigarette aerosols. These data were compared with baseline levels in exhaled breath blanks to place the findings in the context of the known and common presence of some chemical constituents in indoor environments [19,20,21,22]. The analytical methodologies used in this study have been applied to collection and measurement of constituents in exhaled cigarette aerosols [23,24,25,26,27] and have been adapted to measure levels of phenolics and carbonyls in exhaled e-cigarette aerosols.

## 2. Experimental Section

### 2.1. Materials

**Figure 1:**
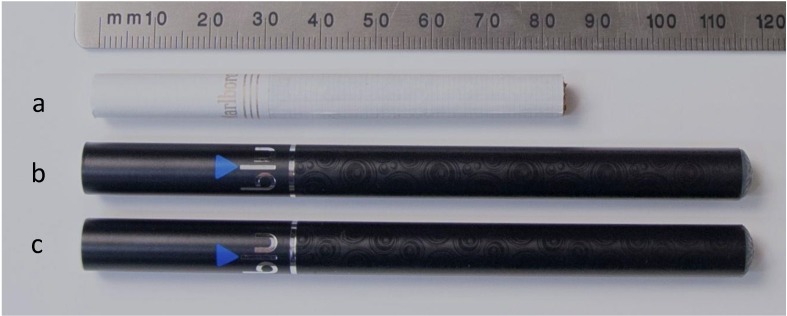
The three study products: (**a**) Marlboro Gold Box, 85 mm conventional cigarette (MGB); (**b**) blu Classic Tobacco Disposabe (blu CTD); (**c**) blu Magnificent Menthol Disposable (blu MMD).

The conventional cigarette and the two e-cigarettes used in this study were all products with significant U.S. market shares in their respective categories. The products used in this study are shown in Figure 1.

The Marlboro Gold King Box filtered cigarette (MGB), which is the largest-selling brand in the U.S. was selected to represent the conventional cigarette category (Philip Morris USA, Miami, FL, USA) [28]. The blu eCigs Classic Tobacco Disposable (blu CTD) and blu eCigs Magnificent Menthol Disposable (blu MMD) electronic cigarettes were selected to represent the e-cigarette category (Charlotte, NC, USA), representing the U.S. market leaders for this product category. The MGB sample was obtained from a commercial wholesaler (Reidsville Grocery Company, 1624 Freeway Dr., Reidsville, NC, USA). The e-cigarette products were obtained directly from the manufacturer.

Both of the disposable e-cigarette products utilize a flow activation design whereby the heating circuit is activated only during puffing. Both e-cigarette products utilize glycerin as the aerosolizing agent and are labeled as containing nicotine (20–24 mg/e-cigarette). Compositions of the e-liquids were 82% glycerin, 9% water, 2% nicotine and 7% flavor for blu CTD; 75% glycerin, 18% water, 2% nicotine and 5% flavor for blu MMD [29]. The e-liquid loadings were 1.03 g and 1.00 g for blu CTD and blu MMD, respectively. Both e-cigarettes utilize 3.7 V batteries, 3.0 Ω atomizers, and both products are designed to deliver approximately 400 puffs.

All three samples were representative of commercially available consumer products at the time of the study. Exhaled aerosols from each of the products were captured on glass fiber filter pads. In addition to the exhaled aerosol from products, exhaled breath blanks were used to establish baseline values for the exhaled cigarette smoke and exhaled e-cigarette aerosol comparisons. Blanks were obtained from each subject prior to the exhaled aerosol sessions by collecting their exhaled breaths.

### 2.2. Experimental Design

This study involved collection of exhaled aerosol from human subjects using conventional cigarettes and e-cigarettes. The experiments were conducted under an IRB-approved protocol (Quorum IRB, 1501 Fourth Ave., Suite 800, Seattle, WA, USA). Subject recruiting was performed by Eastcoast Research (Eastcoast Research, 1118 Grecade St., Greensboro, NC, USA). All sessions were conducted in a 40 m^3^ conference room at the Eastcoast Research facility. Subjects were screened for age (21 ≤ age ≤ 54), product use (e-cigarette subject puffs ≥30 puffs/day; conventional cigarettes >20 cigarettes/day), product preference (MGB, blu CTD or blu MMD) and for a stable preference for the specified products (≥6 months). All subjects were required to abstain from any tobacco product use for a minimum of one hour prior to the collection sessions. Exhaled carbon monoxide levels were verified for the subjects prior to each session with a piCO+ Smokerlyzer (Bedfont Scientific Ltd., Station Road, Harrietsham, Maidstone, Kent ME17 IJA, England) and were required to be less than 10 ppm to participate in the sessions. A total of thirty subjects were recruited for the study—ten subjects for each of the three products.

The three analyte classes (major components, phenolics and carbonyls) studied in this work are listed in Table 1 along with the individual analytes. The major components were selected to provide a mass balance distribution of water, glycerin and nicotine in exhalants from the three products. Some carbonyls have been reported in machine deliveries from e-cigarettes although at levels ten to hundreds of times less than in mainstream cigarette smoke [3,6,11,12]. A recent literature summary of e-cigarette chemical analysis also suggested the presence of *o,m,p*-cresols in the headspace of a single product [30]. Therefore, this work will also establish the levels of carbonyls and phenolics in exhaled aerosols from the cigarette, e-cigarettes and exhaled breaths.

**Table 1:** A listing of the three classes of analytes—major components, phenolic and carbonyl and individual analytes measured in this study.

Analyte Class	Analyte
Major Components	Water
	Glycerin
	Nicotine
Phenolics	Hydroquinone
	Resorcinol
	Catechol
	Phenol
	*m,p*-Cresol
	*o*-Cresol
Carbonyls	Formaldehyde
	Acetaldehyde
	Acetone
	Acrolein
	Propionaldehyde
	Crotonaldehyde
	Methylethylketone
	Butyraldehyde

Total particulate matter, TPM, for three MGB cigarettes and 99 puffs from the two e-cigarettes were all approximately 150 mg under an intense puffing regime [29] and served as the basis for the puffing arrangement in this study. Cigarette subjects used three cigarettes per session and e-cigarette subjects used a maximum of 99 puffs per session. Each subject used their preferred product in a total of nine sessions which provided three replicates per subject in the three analyte classes. Sessions were limited to a maximum of two hours in duration.

### 2.3. Exhaled Collection Method Summary

**Figure 2:**
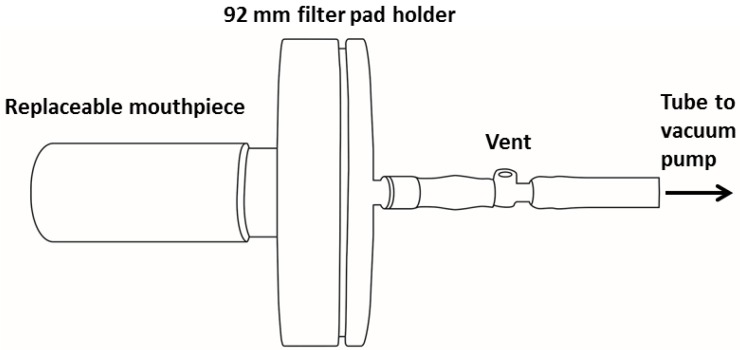
Schematic of the vacuum-assisted collection system for exhaled samples. The single pad collection was used for analysis of phenolics and major components. The apparatus used for the collection of carbonyls included a second filter holder of identical dimensions in series with the first.

This research utilizes modified ISO 17025 accredited conventional cigarette smoke analysis methods to quantitate select analytes in the exhaled aerosols from cigarettes and e-cigarettes. The vacuum-assisted collection system employed in the present work has been previously described [23,24,25,26] and used to quantify a number of different analytes in the exhaled smoke from conventional cigarettes. The system utilizes 92 mm glass fiber filter pads that have greater than 99% efficiency in retaining aerosols in the size range of cigarette smoke, with calibrated vacuum assistance to permit collection of exhaled samples in a manner that is perceived by subjects as neutral in terms of the effort required to deliver exhalate into the collection system. A schematic of the collection system is shown in Figure 2.

The system incorporates a replaceable mouthpiece into which subjects exhale aerosol or breaths. The vacuum pumps were calibrated daily to aspirate 200 mL/min. The tube connecting the pad holder to the vacuum pump was vented to prevent aspiration through the pads when the subjects were not exhaling into the collection system. Subjects covered the vent with a finger when exhaling into the system and then uncovered the vent between exhaled puffs or breaths. A variation of the collection system in Figure 1 was used in carbonyl sessions. Two filter pads arranged in series and treated with a 2,4-dinitrophenylhydrazine (DNPH) solution were used for carbonyl collection sessions to increase sensitivity for these compounds.

#### 2.3.1. Exhaled Breath Blank Collections

Blanks for each participant were collected at the beginning of each session prior to collection of exhaled aerosol from the products. These blanks were performed to obtain baseline levels of analytes in their exhaled breath prior to collection of exhalates from the products. Blanks were collected by instructing the subjects to exhale normal breaths into the vacuum assisted collection system over a twenty-minute period—a maximum of 30 exhaled breaths for cigarette sessions and a maximum of 99 exhaled breaths for e-cigarette sessions.

#### 2.3.2. Carbonyl Room Air Blank Collections

In addition to exhaled breath blanks, a single replicate of room air was sampled with the collection system during each carbonyl session. Carbonyls have been observed in indoor air at levels in excess of 100 µg/m^3^ [19,20,21,22]. Room air background levels of carbonyls were collected in the occupied conference room prior to carbonyl exhaled cigarette and e-cigarette usage sessions. Room air blanks were generated by pulling room air through DNPH treated pads with the vacuum-assisted collection system for 30 simulated exhaled puffs during cigarette sessions and 99 simulated exhaled puffs during e-cigarette sessions. The simulated exhaled puff duration for room air blanks was 2–3 sec.

After completion of the exhaled breath collections, pad holders with new pads were inserted into the collection system and the respective products presented to the subjects. Cigarette smokers were presented with an unopened pack at the beginning of each session and instructed to light their cigarettes, puff normally and exhale their smoke into the collection systems. Similarly, after e-cigarette subjects completing their exhaled breath collections, each subject received a new e-cigarette for the session. Subjects were instructed to take one test puff to verify nominal operation of their test products, puff normally and exhale their aerosol into the collection systems. Pad holders were capped upon completion of the collections and subjected to work-up within 40–60 min.

#### 2.3.3. Analytical Method Capabilities Summary

ISO 17025 methods for cigarette mainstream smoke were verified for use with exhaled aerosol matrices from cigarettes and e-cigarettes. Cartridge-based collections were investigated for carbonyls, but were not suitable for exhaled aerosol collections due to their high resistance to air flow and observed break though during method development. Exhaled aerosol method verification involved spiking and recovery experiments over the response ranges with an emphasis on accuracy and precision at the method limits of quantitation.

A summary of capabilities for the exhaled aerosol methods for e-cigarettes is provided in Table 2 as detection limits, quantitation limits, accuracy and precision. The limit of detection (LOD), is defined as the lowest quantity of an analyte that can be distinguished from the background matrix. The limit of quantitation (LOQ), is the level above which quantitative results may be obtained for an analyte with 99% confidence. Instrument parameters and additional method information for phenolics, carbonyls, glycerin, nicotine and water analyses are available as supplementary materials (Supplemental Files).

**Table 2:** Exhaled aerosol analysis capabilities for major components, phenolics and carbonyls in e-cigarette samples.

Analyte		LOD	LOQ	Accuracy (%)	Precision (%)
Major Components	Nicotine	0.69	4.86	108	2
	Glycerin	0.0059	1.51	101	2
	Water	ND	31	99	0
Phenolics	Hydroquinone	0.37	2.00	113	2
	Resorcinol	0.06	0.40	109	2
	Catechol	0.47	2.00	114	2
	Phenol	0.09	0.32	108	2
	*m,p*-Cresol	0.60	4.00	110	2
	*o*-Cresol	0.16	1.00	113	1
Carbonyls	Formaldehyde	0.10	12.45	97	0
	Acetaldehyde	0.39	5.20	96	1
	Acetone	0.61	13.64	96	3
	Acrolein	0.13	12.34	97	0
	Propionaldehyde	0.21	1.89	98	2
	Crotonaldehyde	0.21	2.17	95	1
	Methylethylketone	0.24	2.06	97	2
	Butyraldehyde	0.18	5.30	95	1

Notes: All units are µg/session except glycerin and water (mg/session). ND—LOD for water was not determined.

## 3. Results and Discussion

### 3.1. Exhaled Aerosol Mass Balance Distribution of Water, Glycerin and Nicotine

The average number of exhaled puffs collected during the water, glycerin and nicotine, phenolic and carbonyl collection sessions were not significantly different between methods as determined by an ANOVA analysis. The average number of exhaled puffs was 30 for three cigarettes and 95 for e-cigarettes during the water, glycerin and nicotine collection sessions.

**Figure 3:**
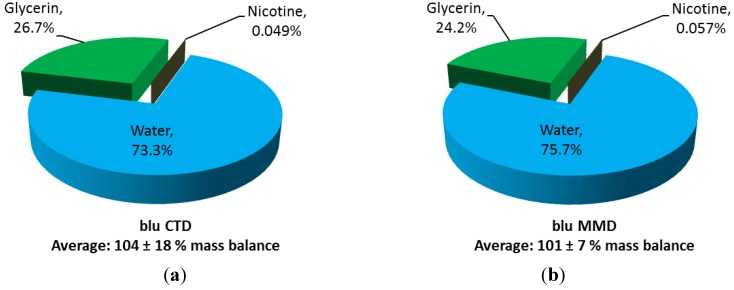
Average distributions and mass balances of water, glycerin and nicotine in exhaled e-cigarette aerosols for (**a**) blu Classic Tobacco Disposable (blu CTD) and (**b**) blu Magnificent Menthol Disposable (blu MMD).

Nicotine, glycerin and water analysis were used to compare distribution and mass balance of these analytes in exhaled aerosols. Distribution is determined by measuring the amounts of these compounds in exhalate collection sessions for the three products and then dividing by the sum total of the three constituents. The average distributions of exhaled e-cigarette aerosols are shown in Figure 3.

The exhaled aerosol mass from the two e-cigarettes is primarily water and glycerin, which together comprise greater than 99.9% of the collected aerosol distribution. Average mass balances for water, glycerin and nicotine were fully accounted for in the e-cigarette aerosols at 104% and 101%. Machine-generated mainstream from e-cigarettes contain approximately 86% glycerin and 8% water [29], which is similar to the e-liquid composition itself. The high concentration of water in the exhaled e-cigarette aerosol has been attributed to water accretion from the respiratory tract by the hydrophilic glycerin aerosol [31].

Average mass balance for nicotine, glycerin and water in exhaled aerosol from the conventional cigarette was (83% ± 21%). The remaining exhaled aerosol mass for cigarettes samples are attributed to particulates from combustion processes known to comprise more than 70% of mainstream conventional cigarette smoke [32,33]. The concentration of nicotine observed in exhaled cigarette smoke was approximately an order of magnitude higher than in the exhaled e-cigarette aerosols (~0.40% *vs.* ~0.05%, respectively). Furthermore, the great majority (~85%) of real-world bystander exposures to nicotine and other smoke constituents in smoking environments is derived from the sidestream smoke emitted from the smoldering cigarette rather than from smokers’ exhaled breaths [14]. Since e-cigarettes do not produce such sidestream emissions, the reductions in most potential bystander chemical exposures that accompany indoor e-cigarette usage as opposed to smoking may be anticipated to be even greater than the differences in exhaled nicotine concentrations of the very different aerosols. The public health impacts of environmental tobacco smoke have been overwhelmingly attributed to chemical constituents other than nicotine, so the simple presence of some nicotine in the exhalate of e-cigarette users does not suggest a basis for concern about bystander exposures.

### 3.2. Exhaled Phenolics and Carbonyls

The majority of phenolic and carbonyl measurements in exhaled e-cigarette aerosols were either not detectable, below the detection limits or below the quantitation limits. However, these analytes were consistently observed in exhaled cigarette smoke at quantifiable levels. Example data are shown in Table 3 for hydroquinone and acetaldehyde.

**Table 3:** Hydroquinone and acetaldehyde in exhaled aerosol (µg/session) for Marlboro Gold Box (MGB), blu Classic Tobacco Disposable (blu CTD) and blu Magnificent Menthol Disposable (blu MMD).

MGB			Blu CTD			Blu MMD		
Subject	Acetaldehyde	Hydroquinone	Subject	Acetaldehyde	Hydroquinone	Subject	Acetaldehyde	Hydroquinone
1	227.6	70.6	11	<LOQ	<LOD	21	16.7	<LOD
	186.0	60.0		<LOQ	<LOD		35.3	<LOD
	221.0	69.1		<LOQ	<LOD		38.9	<LOD
2	134.7	41.3	12	<LOQ	<LOD	22	<LOQ	<LOD
	129.8	33.2		<LOQ	<LOD		<LOQ	<LOD
	107.7	31.9		<LOQ	<LOD		<LOQ	<LOD
3	131.2	32.2	13	<LOQ	<LOD	23	<LOQ	<LOD
	169.0	47.4		86.4	<LOD		<LOQ	<LOD
	128.1	52.5		44.2	<LOD		<LOQ	<LOD
4	115.6	48.5	14	<LOQ	<LOD	24	5.4	<LOD
	119.3	47.3		<LOQ	<LOD		7.2	<LOD
	124.1	42.5		<LOQ	<LOD		9.9	<LOD
5	195.4	18.4	15	<LOQ	<LOD	25	<LOQ	<LOD
	122.0	13.3		<LOQ	<LOD		<LOQ	<LOD
	196.3	20.0		<LOQ	<LOD		<LOQ	<LOD
6	208.0	99.5	16	<LOQ	<LOD	26	<LOQ	<LOD
	116.9	103.5		<LOQ	<LOD		<LOQ	<LOD
	116.0	83.9		<LOQ	<LOD		<LOQ	<LOD
7	<LOQ	22.8	17	<LOQ	<LOD	27	<LOQ	<LOD
	88.1	8.79		<LOQ	<LOD		<LOQ	<LOD
	48.1	25.9		<LOQ	<LOD		6.2	<LOD
8	380.2	29.1	18	<LOD	<LOD	28	<LOQ	<LOD
	193.7	37.7		24.2	<LOD		<LOQ	<LOD
	189.7	30.9		<LOQ	<LOD		7.1	<LOD
9	285.2	73.0	19	<LOQ	<LOD	29	6.5	<LOD
	126.6	26.8		<LOQ	<LOD		8.9	<LOD
	104.6	81.6		<LOQ	<LOD		7.6	<LOD
10	217.6	43.0	20	6.9	<LOD	30	<LOQ	<LOD
	162.7	46.2		<LOQ	<LOD		<LOQ	<LOD
	114.1	64.0		<LOQ	<LOQ		5.4	<LOD
Avg *	156.7	46.8		<9.73 *	<0.421 *		<8.29 *	<0.367 *
*SD*	68.8	24.7		16.5	0.3		8.2	0.0
*LOQ*	41.6	2.00		5.20	2.00		5.20	2.00
*LOD*	0.390	0.367		0.390	0.367		0.390	0.367

Note: * LOD and LOQ values were averaged to provide upper limit estimates in exhalates from the two e-cigarette samples.

To simplify data reporting, total phenolic compounds and total carbonyl compounds in exhaled aerosols are presented for each product, along with exhaled breath blanks for comparison. Upper-limit estimates for exhaled aerosol compositions are accomplished by using the method limits for observations below the limits of detection and quantitation. In cases where individual measurements were less than the limits of quantitation, the limit of quantitation values were used and in cases where the measurements were non-detects or less than the limits of detection, the limit of detection values were used to compare analytes in exhaled aerosol between products. ANOVA comparisons were performed to test for differences between exhaled aerosol samples, breath blanks and room air (α = 0.05).

**Figure 4:**
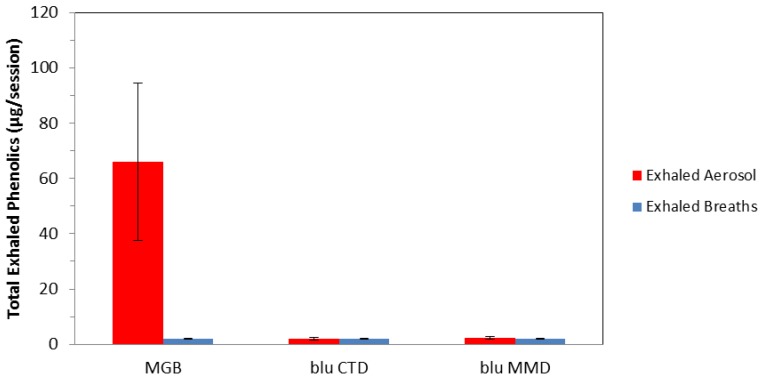
Total exhaled phenolics for exhaled aerosol and breaths for Marlboro Gold Box (MGB), blu Classic Tobacco Disposable (blu CTD) and blu Magnificent Menthol Disposable (blu MMD).

Total exhaled phenolics are shown in Figure 4 for exhaled aerosol and breaths collected following use of each product. The average number of exhaled puffs was 29 for three cigarettes and 98 for e-cigarettes during the phenolics collection sessions. Phenolics in exhaled breath blanks were all below limits of quantitation or limits of detection for the three products tested. The average total phenolics in exhaled e-cigarette aerosols were not statistically different than in exhaled breaths. In contrast, the average total phenolic compounds in exhaled smoke for cigarette subjects averaged 66 µg/session and ranged from 36 to 117 µg/session, significantly greater than in exhaled e-cigarette aerosol or exhaled breaths. The total phenolics for the ten MGB subjects is comparable, although higher, than data reported by Moldoveanu [23] for the phenolic compounds reported here, (12.3 µg/3 cigs, range 6–25 µg/3 cigs).

**Figure 5:**
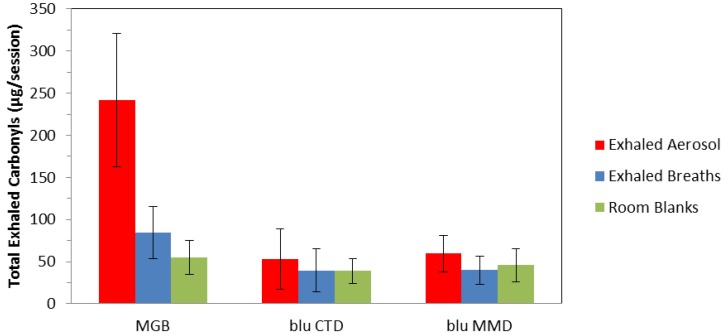
Total carbonyls in exhaled aerosol, breaths and room blanks for Marlboro Gold Box (MGB), blu Classic Tobacco Disposable (blu CTD) and blu Magnificent Menthol Disposable (blu MMD).

Figure 5 summarizes total carbonyl compounds exhaled from each product, exhaled breaths and room blanks. The average number of exhaled puffs was 27 for three cigarettes and 98 for e-cigarettes during the carbonyl collection sessions. Carbonyls in room air blanks and exhaled breath blanks were observed at the levels of quantitation due to the pervasive nature of carbonyls in indoor environments [20,21,22,23]. Room air blanks, exhaled breath blanks and exhalates from the two e-cigarettes were not statistically different. And as a result, total carbonyls in exhalates from the two e-cigarettes were not distinguishable from exhaled breaths or room air blanks. However, total carbonyls in exhaled smoke from cigarettes were significantly greater than the total carbonyls in exhaled e-cigarette aerosols, exhaled breaths and room blanks (average 242 µg/session, range 136–352 µg/session). The total carbonyls for the ten MGB subjects is comparable to historical data from Moldoveanu [24], for the carbonyls reported here, (average 183 µg/3 cigs, range 122–309 µg/3 cigs).

The absence of carbonyls and phenolics at quantifiable levels in exhaled e-cigarette aerosols is also demonstrated by comparing acetaldehyde and hydroquinone, as examples, for exhaled aerosol from products, breath blanks and room air as shown in Table 4. The sample aerosol values for the e-cigarettes are not statistically different than breath blanks, or room blanks.

**Table 4:** Hydroquinone and acetaldehyde in exhaled aerosol, breaths and room air (µg/session) for blu Classic Tobacco Disposable (blu CTD) and blu Magnificent Menthol Disposable (blu MMD).

Analyte		Blu CTD			Blu MMD		
		Aerosol	Breaths	Air	Aerosol	Breaths	Air
Hydroquinone	Mean	<0.421 *	<0.367 *	ND	<0.367 *	<0.367 *	ND
	SD	0.3	0.0	ND	0.0	0.0	ND
Acetaldehyde	Mean	<9.73 *	<9.58 *	<3.60 *	<8.29 *	<5.20 *	<5.20 *
	SD	16.5	16.0	2.3	8.2	0.0	0.0

Note: * LOD and LOQ values were averaged to provide upper limit estimates in the aerosol, breath and air samples. ND—Room air blanks were not determined for phenolics.

Recent work by Robinson, *et al.* characterized the potential for second-hand e-cigarette exposure in indoor air from human subjects using validated air sampling methods (ASTM, EPA, NIOSH and OSHA) for 34 HPHC analytes [34]. Carbonyls and phenolics were no different than background levels in the room when the study subjects used e-cigarettes. Carbonyls were significantly greater than background when conventional cigarettes were smoked. Phenolics were no different than background for conventional cigarettes. Combustion byproducts were not observed above background for e-cigarettes but were present during conventional cigarette use.

The findings of this study establish the substantial reduction in the complexity and quantities of select chemical constituents in exhaled aerosols from e-cigarettes relative to exhaled smoke from conventional cigarettes. These constituents are expected in mainstream and exhaled conventional cigarette smoke as demonstrated in this study and in extant literature since their formation is a result of combustion and pyrolysis processes. However, the thermal vaporization mode of operation common to e-cigarette designs does not provide a combustion formation pathway for those analytes. Whereas the present work has focused on the smaller, cigarette-like devices that have historically been market leaders in the U.S., the operation of these devices is fundamentally very similar to that of the larger, tank-style products that are increasingly favored by vapers in the U.S. and elsewhere around the world. The emerging technical literature in this area is consistent with an expectation that similarities in emitted and exhaled aerosols across the spectrum of innovative new e-cigarette designs will continue to demonstrate markedly reduced exposures to both users and bystanders relative to those that occur from conventional cigarette smoking.

## 4. Conclusions

This study was designed to measure phenolics and carbonyls in exhaled cigarette smoke, exhaled e-cigarette aerosols and exhaled breaths using a vacuum-assisted, pad collection system. This collection system was also used to determine a mass balance and distribution for water, glycerin and nicotine in exhaled e-cigarette aerosol. Distribution of exhaled e-cigarette aerosol showed the composition was greater than 99.9% water and glycerin, a small amount of nicotine (<0.06%) and gave a quantitative mass balance for these analytes in the exhaled aerosol mass, (101%–104%). Exhaled aerosol collections from e-cigarettes averaged over three times more exhaled puffs than from the conventional cigarettes. Total phenolics in exhaled e-cigarette aerosol were not significantly different than the amounts observed in exhaled breaths. Total phenolics in exhaled cigarette smoke were greater than in exhaled breaths and averaged 66 µg/session for the test subjects. Similar results were observed for carbonyl compounds in exhaled aerosols. Total carbonyls in exhaled e-cigarette aerosol were not significantly different than those in exhaled breaths and room air blanks. Carbonyls in exhaled cigarette smoke were greater than in exhaled breaths, room air blanks and exhaled e-cigarette aerosols, with an average total carbonyl content of 242 µg/session for the cigarette test subjects. Exhaled phenolics and carbonyls in cigarette smoke were comparable to historical data, although higher for the phenolics class in the present study than in prior work. The findings of this work suggest that exhaled e-cigarette aerosol does not increase bystander exposure for phenolics and carbonyls above the levels observed in exhaled breaths of air, in contrast to the quantifiable levels of these analytes in exhaled conventional cigarette smoke.

## Supplementary Files

Supplementary File 1
