# Supplementary material for: Comparison of Select Analytes in Exhaled Aerosol from E-Cigarettes with Exhaled Smoke from a Conventional Cigarette and Exhaled Breaths
Source: Int J Environ Res Public Health. 2014 Oct 27;11(11):11177–91. doi: 10.3390/ijerph111111177 (PMC4245607; doi:10.3390/ijerph111111177)
Supplement: Supplementary File 1 [file ijerph-11-11177-s001.pdf]

*Supplementary Information*

# Comparison of Select Analytes in Exhaled Aerosol from E-cigarettes with Exhaled Smoke from a Conventional Cigarette and Exhaled Breaths

## 1. Glycerin, Nicotine and Water

Masses of 92 mm filter pad holders were determined before and after the collection sessions. The pads were transferred to 250 mL amber vials and then charged with 60 mL of 0.1% anethole V/V in methanol (LabChem Inc., 200 William Pitt Way, Pittsburg, PA 15238, USA) within 15 min of completing the collection session. Extraction was accomplished with mechanical wrist action shaking for a minimum of 35 min. Glycerin (99.7%, Vitusa Products Inc., 343 Snyder Ave., Berkley Heights, NJ 07922, USA) was quantitated with an Agilent 6890 GC-FID (Agilent Technologies, 5301 Stevens Creek Blvd., Santa Clara, CA 95051, USA). Separation of the 1.0 μL injections were achieved with a DB-WAX (15 m × 0.25 mm i.d. × 0.25 μm film) capillary column. A double taper injection liner with glass wool was held at 300 °C with a split ratio of 40, a pressure equilibration time of 0.5 min and gas saver flow of 20.0 mL/min for 19.0 min. The FID detector was held at 300 °C with the make up gas flow comprising helium (50.0 mL/min), air (450 mL/min) and hydrogen (40 mL/min). The oven and pressure parameters were as follows: 0.0 min = 0.8 psi, 60 °C; 0.5 min = 0.8 psi, 60 °C, 4.5 min = 0.8 psi, 200 °C, 4.9 min = 1.2 psi, 240 °C, 6.8 min = 1.2 psi, 240 °C.

Nicotine (99.0%, Alchem International Ltd., Main Mathura Rd., Village Kaili, Ballabgarh, Faridabad-121004, Haryana India) was quantitated with an Agilent 6890 GC-MS using a 15 m VF-WAXms (15 m × 0.25 mm i.d. × 0.25 μm film) column. Injection volume was 1.0 μL into a Sky 4.0 mm single taper inlet liner held at 300 °C, splitless. A solvent delay of 3 min was used. The initial oven temperature of 65 °C increased up to 240 °C by a rate of 20 °C/min with helium as carrier gas at a constant flow of 1 mL/min. The total run time was 9.0 min; solvent delay: 3 min; MSD transfer line: 250 °C, ion source temperature: 230 °C, MS quad temperature 150 °C; acquisition mode: SIM with m/z = 84.0, 133.0.

Aliquots of the same samples analyzed for nicotine and glycerin were also analyzed for water content. Water was measured by a standardized Karl Fisher method [1] with the extraction volume reduced to 60 mL. A weighed aliquot of approximately 5 mL was injected into the reaction vessel of a Metrohm 901 Titrando™ (Metrohm USA, Inc., 6555 Pelican Creek Circle, Riverview, FL 33578, USA), and titrated using Hydranal™ Composite 5 (5 mg water/mL) using a weighed, airtight syringe (Hamilton Company, 4970 Energy Way, Reno, NV 89502, USA; model #1725). The titer value for the Hydranal™ solution was 4.85 mg water/mL. The system performed the titration until a rate equal to or less than 20 μL/min was reached. The amount of water collected on the filter pad was based on the volume of Hydranal™ solution required to reach the end point and the titer value. The limit of quantitation was determined to be 20 mg water/session for exhaled cigarette smoke and 31 mg water/session for exhaled e-cigarette aerosol. Limit of detection was not determined for water analysis.

## 2. Phenolics

Phenolics in exhaled samples were quantitated with a method based upon ISO accredited methodology for cigarette mainstream smoke. Weighed 92 mm filter pad holders from collection sessions were disassembled and the pads transferred to 40 mL amber vials. The pads were extracted with 20 mL of 1% acetic acid/2.5% methanol/Type 1 water on a mechanical wrist action shaker for 30 min. An aliquot was filtered through a 2 µm polypropylene filter (Whatman® Puradisc 25 PP, Sigma-Aldrich, P.O. Box 14508, St. Louis, MO 63178, USA). The filtered sample (2 µL) was injected into an Aquity UPLC (Waters Corporation, 34 Maple Street, Milford, MA 01757, USA) using a KinetexTC18 column (150 mm × 2.1; 1.7 µm). A flow rate of 0.4 mL/min and a gradient run beginning with 98:2 1% acetic acid/Type 1 water and 1% acetic acid/1% isopropanol/acetonitrile is used to separate the compounds. Phenols are detected with a fluorescence detector set to an excitation wavelength of 304 nm and an emission wavelength of 338 nm. At 1.9 min. the excitation changes to 274 nm and the emission goes to 298 nm. Phenol standards were purchased as a custom mix from Sigma-Aldrich (Lot No: LC00767) and were diluted in 1% acetic acid/100 mM ascorbic acid/Type 1 water.

## 3. Carbonyls

Carbonyls in exhaled samples were quantitated based upon a modified method used by Moldoveanu for exhaled cigarette smoke [2] and a standardized method for mainstream cigarette smoke [3]. Two collection pads, in series, were used to enhance collection of carbonyls at low levels. The pad holder configuration for carbonyl analysis was similar to that depicted in Figure 1, with an additional filter holder and collecting filter pad installed in series immediately after the first filter pad collecting unit as shown below in Figure S1.

**Figure S1.** Schematic of the two filter pads in series used to collect exhaled and room air samples for carbonyl analyses.

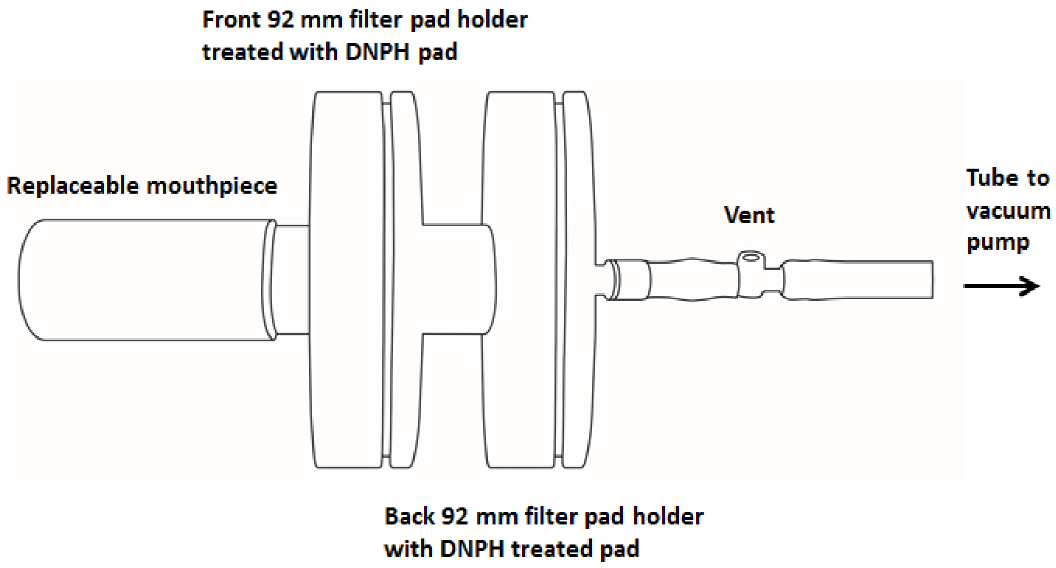

To each 92 mm pad, 8.3 mL of a DNPH (Sigma-Aldrich; 42210-100G-F) pad solution consisting of 15.0 mg/mL DNPH in acetonitrile (VWR International, Radnor Corporate Center, Building One, Suite 200, P.O. Box 6660, 100 Matsonford Road, Radnor, PA 19087, USA, B&J; Carbonyl-free grade; Cat. 018-4) and 2.5 µL/mL of 85% phosphoric acid (Mallinckrodt Pharmaceuticals, 675 McDonnell Blvd, St. Louis, MO 63042, USA, No. 2796-05) was added. The pads were allowed to air dry in a hood until the masses were reduced to between 0.4 to 0.8 g above the pad dry weights. The DNPH pads were stored in a dark desiccator containing a small amount of acetonitrile. The DNPH treated pads were prepared less than 24 hours prior to placement in the pad holders.

The front and back pads were allowed to sit in capped pad holders between 40 and 60 minutes after the collection sessions were completed. Both pads were then placed in a 60 mL vial containing 50 mL of acetonitrile. The vials were shaken for approximately 30 minutes by mechanical wrist action. A 4 mL aliquot was transferred to a 20 mL vial containing 0.33 mL of aqueous Trizma base, 39.0 mg/mL (Sigma; T6066-100G). The vial was shaken briefly and filtered into an LC injection vial using a syringe equipped with 0.45 µm TF and 0.2 µm PP (Whatman Puradisc Cat. 6785-250 and Cat. 6788-2502, respectively) tandem filter discs. The filtered sample (2 µL) was injected into an Acuity UPLC using a TBEH C18 column (100 × 2.1 mm; 1.7 um) with PDA detector at 355 nm. The mobile phases were Type 1 water and acetonitrile, carbonyl-free. Neat materials of individual DNPH derivatized standards were purchased from Chem Service (660 Tower Lane, P.O. Box 599, West Chester, PA 19281, USA) and prepared in acetonitrile.

## References

1. CORESTA Recommended Method N° 15 Cigarettes-Determination of Water in Smoke Condensates. Karl Fischer Method (March 1990). Available online: http://www.coresta.org/Recommended_Methods/CRM_15.pdf (accessed on 20 October 2014).
2. Moldoveanu, S.; Coleman, W., III; Wilkin, J. Determination of carbonyls in exhaled cigarette smoke. *Beitr. Tabakforschung Int.* **2007**, *22*, 346–357.
3. CORESTA Recommended Method N° 74 Determination of Selected Carbonyls in Mainstream Cigarette Smoke by HPLC (July 2014). Available online: http://www.coresta.org/Recommended_Methods/CRM_74-update%28July14%29.pdf (accessed on 20 October 2014).

© 2014 by the author; licensee MDPI, Basel, Switzerland. This article is an open access article distributed under the terms and conditions of the Creative Commons Attribution license (http://creativecommons.org/licenses/by/4.0/).
